# Feasibility study of on-site solid-state enzyme production by *Aspergillus oryzae*

**DOI:** 10.1186/s13068-020-1669-3

**Published:** 2020-02-26

**Authors:** Satoru Shinkawa, Shigenobu Mitsuzawa

**Affiliations:** 1 Fundamental Technology Center, Honda R&D Co., Ltd., 1-4-1 Chuo, Wako-shi, Saitama, 351-0113 Japan; 2 Present Address: Honda Research Institute Japan Co., Ltd., 8-1 Honcho, Wako-shi, Saitama, 351-0188 Japan

**Keywords:** On-site enzyme production, Solid-state fermentation, *Aspergillus oryzae*, Ammonia-treated rice straw, Acetic acid, *pyrG*, *ligD*, Cellobiohydrolase, β-Glucosidase, Endoxylanase

## Abstract

**Background:**

The development of biorefinery systems that use lignocellulosic biomass as a renewable carbon source to produce fuels and chemicals is attracting increasing attention. The process cost of enzymatic saccharification of biomass is a major challenge for commercialization. To decrease this cost, researchers have proposed on-site solid-state fermentation (SSF). This study investigated the feasibility of using *Aspergillus oryzae* as a host microorganism for SSF recombinant enzyme production with ammonia-treated rice straw as model biomass. Eight *A. oryzae* strains were tested, all of which are used in the food industry. We evaluated the effects of acetic acid, a fermentation inhibitor. We also developed a platform strain for targeted recombinant enzyme production by gene engineering technologies.

**Results:**

The SSF validation test showed variation in the visibility of mycelium growth and secreted protein in all eight *A. oryzae* strains. The strains used to produce *shoyu* and *miso* grew better under test conditions. The ammonia-treated rice straw contained noticeable amounts of acetic acid. This acetic acid enhanced the protein production by *A. oryzae* in a liquid-state fermentation test. The newly developed platform strain successfully secreted three foreign saccharifying enzymes.

**Conclusions:**

*A. oryzae* is a promising candidate as a host microorganism for on-site SSF recombinant enzyme production, which bodes well for the future development of a more cost-efficient saccharifying enzyme production system.

## Background

The development of biorefinery systems that use lignocellulosic biomass as a renewable carbon source to produce fuels and chemicals is attracting increasing attention because of the social need for replacing fossil fuel resources and decreasing carbon dioxide emissions [1, 2]. Since lignocellulosic biomass (e.g., corn stover and rice straw) do not compete with the food supply, unlike agricultural crops (e.g., sugarcane and corn), they are considered promising sustainable feedstocks [3]. Especially, rice straw is attracting attention as a bioethanol production resource in Asian countries, including Japan [4]. The conversion of lignocellulosic biomass into fuels and chemicals involves hydrolysis of the plant cell wall into fermentable sugars, which are, in turn, converted to fuels or chemicals [5, 6]. In contrast to starch-based biomass, the lignocellulosic plant cell wall has a much recalcitrant structure in which crystalline cellulose is surrounded by hemicellulose and lignin; therefore, the process cost associated with lignocellulosic biomass saccharification is a major challenge for commercialization [7, 8]. In most general systems, after pretreatment of feedstock biomass, saccharifying enzymes are used as catalysts for hydrolysis of the plant cell wall [9]. Various pretreatment approaches have been studied, which can be categorized into physicochemical methods, including acid, alkaline, hydrothermal and ionic liquid, and biological methods, including microbial and enzymatic delignification [10]. Among these, alkaline pretreatment is one of the most promising process options primarily because of its effectiveness and relatively simple process scheme [11]. Our group has focused on the process that uses aqueous ammonia as an active reagent. The main effects of the pretreatment on herbaceous biomasses are that it selectively dissolves lignin and hemicellulose, cleaves intermolecular ester bonds without degrading carbohydrates, and increases porosity and surface area accessible to saccharifying enzymes, which have been confirmed using the gas sorption method [12]. In the neutralization of the processed biomasses, ammonia can be easily removed by evaporation, because it is highly volatile. Moreover, we have confirmed the efficiency of the ammonia recycling system in a pilot-scale plant [13]. Residual ammonia can be neutralized by acid chemicals, such as sulfuric acid, and can be used as a nutrient by fermenting microorganisms growing on the pretreated biomasses or their hydrolysates in a subsequent process. These versatile processing options of aqueous ammonia provide strong advantages compared with other alkaline reagents.

Since enzymatic saccharification can occur under ambient conditions of temperature and pressure, where electricity or gas expenses are relatively small or low, the process cost is almost equivalent to the enzyme cost, except capital cost [14]. As the enzyme cost is the product of (i) the amount of enzyme used and (ii) the production cost of enzyme per unit, it can be decreased by separately lowering these two factors. First, to decrease the amount of enzyme used, we constructed synthetic mixtures of three cellulase components from *Talaromyces cellulolyticus* and two additive hemicellulases from different organisms to enhance the specific activity per weight [15, 16]. To further decrease the amount of enzyme used, we also examined the amino acid mutations on *T. cellulolyticus* cellobiohydrolase (CBH), which was the most abundant component in the synthetic mixture, exhibiting higher activity compared with the wild type (WT) [17].

**Fig. 1:**
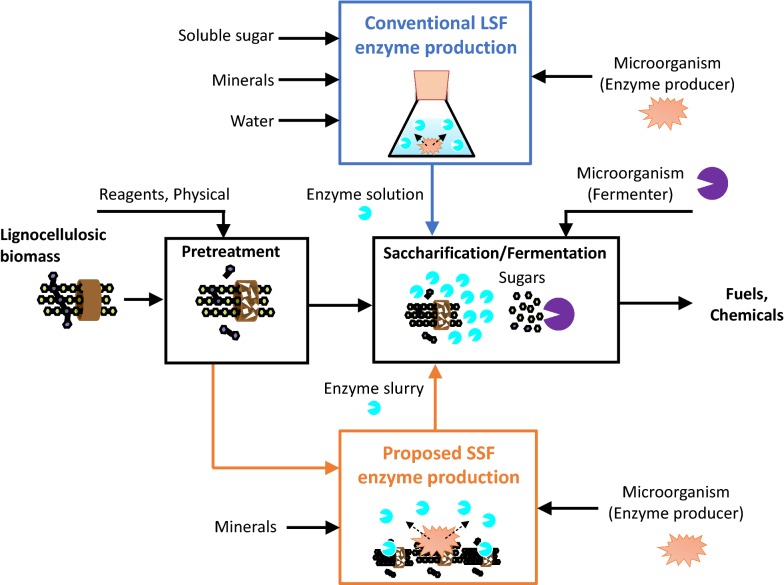
Two methods of on-site enzyme production annexed to the main process line of biomass utilization. (Top) Conventional LSF. (Bottom) Proposed SSF. LSF, liquid-state fermentation; SSF, solid-state fermentation

In parallel, to decrease the production cost of enzyme per unit, on-site enzyme production is desirable where enzyme production is annexed to the main process line of biomass pretreatment, saccharification, and fermentation [18, 19]. There are two methods of on-site enzyme production (Fig. 1). In the conventional method, soluble sugars obtained as by-products in food manufacturing (e.g., molasses and corn steep liquor) are used as nutrients (i.e., carbon sources) for culturing enzyme-producing microorganisms. Therefore, enzyme fermentation occurs in the liquid state (liquid-state fermentation [LSF]) [20–22]. This method enables the mass production of enzymes of uniform quality. In contrast, researchers including Marx et al. [23] and Mitsuzawa et al. [24] proposed a different production process. Since the aim is to produce enzymes for biomass saccharification in the main process line, it is conceivable to use biomass as the nutrient for enzyme-producing microorganisms. In this method, the biomass used is solid, so enzyme fermentation occurs in the solid state (solid-state fermentation [SSF]). With regard to a decrease in the enzyme cost, SSF has potentially two advantages over conventional LSF: first, SSF can do away with nutrient expense. Second, SSF requires much less water, and it is possible to downsize fermentation tanks for on-site enzyme production, leading to a decrease in capital depreciation. Notably, in a cost estimation done by the National Renewable Energy Laboratory (NREL) for LSF, nutrient expense and the capital depreciation were the top cost factors, comprising 78% of the total cost (nutrient expense, 57%; capital depreciation, 21%) [14].

Although theoretical advantages of the SSF enzyme production system are appreciated, a few studies have been compared with the conventional LSF system. To realize the SSF system, we need to use a suitable microorganism that secretes saccharifying enzymes and grows on a given biomass in the solid state. *Aspergillus oryzae* is an important filamentous fungus used in the Japanese food industry, and is also used in the production of *sake* (rice wine), *shoyu* (soy sauce), and *miso* (soybean paste) [25, 26]. Because of its long history of extensive use in the food industry, the US Food and Drug Administration (FDA) has placed *A. oryzae* on the list of Generally Recognized as Safe (GRAS) organisms [27]. In food production, *A. oryzae* secretes large amounts of amylase and protease on solid-state substrates (e.g., steamed rice, wheat, and beans). The efficiency of enzyme production increases in SSF compared with that in LSF conditions [28]. Since Machida et al. [29] published the genomic sequence of the *A. oryzae* strain RIB40 in 2005, various basic genome and metabolome studies have been conducted using this strain, making it one of the most extensively used strains of *A. oryzae* [30–32]. In parallel, numerous powerful and useful genetic engineering techniques have been established for *A. oryzae*: autonomously replicating plasmid vector [33], positive selection of a transformant using auxotrophic strains (e.g., the orotidine-5′-decarboxylase gene [*pyrG*]-deficient strain that requires uridine [34–37]), a marker-recycling system using self-homologous recombination of genomic DNA [38, 39], and an efficient gene-targeting system based on *ligD* deletion, which is involved in nonhomologous end joining [40]. The genetic toolset has made it possible to engineer *A. oryzae* strains for the production of foreign recombinant enzymes [41–43]. On the basis of both the application field and scientific background, *A. oryzae* is considered a good candidate microorganism for on-site SSF recombinant enzyme production.

In this feasibility study, first, we performed a validation test on eight *A. oryzae* strains, all of which are used in the food industry and grown on ammonia-treated rice straw [12, 13]. The visibility of mycelium growth and secreted protein were assessed to examine the feasibility of the proposed on-site SSF recombinant enzyme production system. Because ammonia-treated rice straw contains noticeable amounts of acetic acid, a fermentation inhibitor, we evaluated the effect of acetic acid [44, 45] on the protein production by the selected *A. oryzae* strains. Second, we developed a platform strain for targeted recombinant enzyme production in SSF. We sequenced the entire genome of a selected *A. oryzae* strain and, using the data, obtained a *pyrG*- and *ligD*-deficient *A. oryzae* strain. Third, we tested the transformation of the platform *A. oryzae* strain with three foreign saccharifying enzyme genes and successfully confirmed secretion of the enzymes.

## Results

### Preparation of pretreated biomass

Table 1 summarizes the contents of polysaccharides and chemical compounds reported as fermentation inhibitors in the ammonia-treated rice straw used in this study; the data for dilute sulfuric acid-treated corn stover reported previously [46] are also shown for comparison. Although the glucan content of the two types of biomass was comparable, ammonia-treated rice straw had more than twice xylan content compared to dilute sulfuric acid-treated corn stover. The content of all five fermentation inhibitors (i.e., acetic acid, formic acid, vanillin, 5-hydroxymethylfurfural [HMF], and furfural) was less in ammonia-treated rice straw compared to dilute sulfuric acid-treated corn stover: The content of the two organic acids tested (i.e., acetic acid and formic acid) was about half, whereas that of the three aromatics (i.e., vanillin, HMF, and furfural) was less than a few percentage points.

**Table 1:** Contents of polysaccharides and potential fermentation inhibitors in the pretreated biomasses

Pretreated biomass	Polysaccharide (% biomass)		Potential fermentation inhibitor (mg/g biomass)				
	Glucan	Xylan	Acetic acid	Formic acid	Vanillin	HMF	Furfural
Ammonia-treated rice straw (present study)	35	17	8.5	1.1	0.045	< 0.005	0.019
Dilute sulfuric acid-treated corn stover Zhang et al. [46]	39	7	16.65	1.97	1.27	3.38	5.13

### Validation test of *A. oryzae* strains in SSF using pretreated biomass

**Fig. 2:**
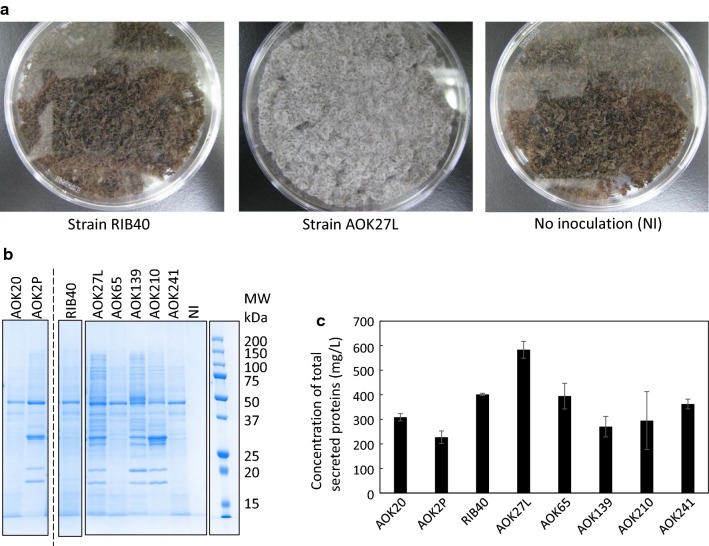
Validation test of *Aspergillus oryzae* strains under SSF conditions using ammonia-treated rice straw. **a** Images of biomass inoculated with *A. oryzae* strains RIB40 and AOK27L and without inoculation (NI). **b** SDS-PAGE of the extractions from solid-state cultures of the eight *A. oryzae* strains tested and uninoculated biomass (NI). **c** Amount of total secreted protein for the eight *A. oryzae* strains tested. Error bars represent the standard deviations of duplicates. SSF, solid-state fermentation; SDS-PAGE, sodium dodecyl sulfate-polyacrylamide gel electrophoresis

Table 2 lists the eight *A. oryzae* strains tested in the SSF experiment to determine whether they can potentially serve as saccharifying enzyme producers, growing on ammonia-treated rice straw, categorized into three groups according to their original industrial purposes. After incubation for 40 h, apparent mycelium growth was confirmed by visual inspection for *A. oryzae* strains AOK2P, AOK210, AOK27L, and AOK139 (Table 2, Fig. 2a and Additional file 1: Fig. S1). Mycelium was not clearly visible for the other four strains, but the particles of the inoculated biomasses were observed to coagulate with one other, which suggested minimal growth of the tested strains. Uninoculated biomass did not exhibit this coagulated form.

**Table 2:** *Aspergillus oryzae* strains tested in the validation test in SSF condition

Group	Industrial purpose	Strain	Visibility of mycelium
1	*Sake* (Rice wine)	RIB40	No
		AOK20	No
		AOK2P	Yes
		AOK65	No
		AOK241	No
2	*Shoyu* (Soy sauce)	AOK210	Yes
3	*Miso* (Soybean paste)	AOK27L	Yes
		AOK139	Yes

Visibility of mycelium indicates whether mycelium was confirmed by visual inspection in the validation test

Sodium dodecyl sulfate-polyacrylamide gel electrophoresis (SDS-PAGE) analysis of the extractions obtained by washing solid-state cultures confirmed that all eight *A. oryzae* strains secreted a substantial amount of proteins (Fig. 2b). Each strain also expressed a characteristic band pattern. The amount of total proteins secreted was evaluated for each *A. oryzae* strain (Fig. 2c); the *A. oryzae* strain AOK27L yielded the largest amount, which could be attributed to good growth. These results confirmed that *A. oryzae* can grow on ammonia-treated rice straw and secretes proteins under SSF conditions.

### Effect of acetic acid on protein

Under SSF conditions for the above validation experiment, the water content was adjusted to 50% (i.e., equal to the weight of dry pretreated biomass), and autoclaving for sterilization did not affect this value significantly. Under the two assumptions that (i) during cultivation, the water content would stay stable ~ 50% in an environmental chamber whose relative humidity was set at 95% and (ii) the reported fermentation inhibitors of the compositions in the pretreated biomass (Table 1) would totally elute to the aqueous phase, the concentration of each fermentation inhibitor in the aqueous phase during the experiment was estimated as follows: 8.5 g/L of acetic acid, 1.125 g/L of formic acid, 0.045 g/L of vanillin, < 0.005 g/L of HMF, and 0.019 g/L of furfural.

**Fig. 3:**
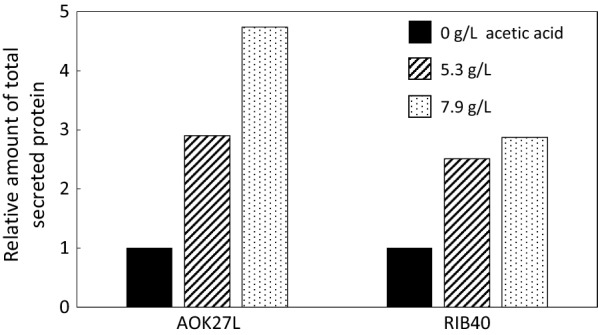
Effects of acetic acid on protein production by *Aspergillus oryzae* strains AOK27L and RIB40

To further examine the influence of acetic acid on enzyme production, the total amount of secreted protein by the two *A. oryzae* strains AOK27L, which yielded the largest amount of total proteins secreted in the SSF experiment (Fig. 2c), and RIB40, which is one of the most extensively studied strains of *A. oryzae,* were assessed in PD liquid medium (pH 6) containing 0, 5.3, or 7.9 g/L acetic acid. We found that 5.3 and 7.9 g/L of acetic acid increased the total amount of proteins secreted by 2.9 and 4.7 times, respectively, for *A. oryzae* strain AOK27L and by 2.5 and 2.9 times, respectively, for *A. oryzae* strain RIB40 (Fig. 3).

### Construction of a platform strain for recombinant enzyme production

Starting from the selected WT *A. oryzae* strain AOK27L, we developed a strain that would serve as a platform for targeted recombinant enzyme production in SSF. Prior to genetically engineering the *A. oryzae* strain, we sequenced its genomic DNA. Using these data, we designed the plasmid constructs and PCR primers (see "Methods" for details).

**Fig. 4:**
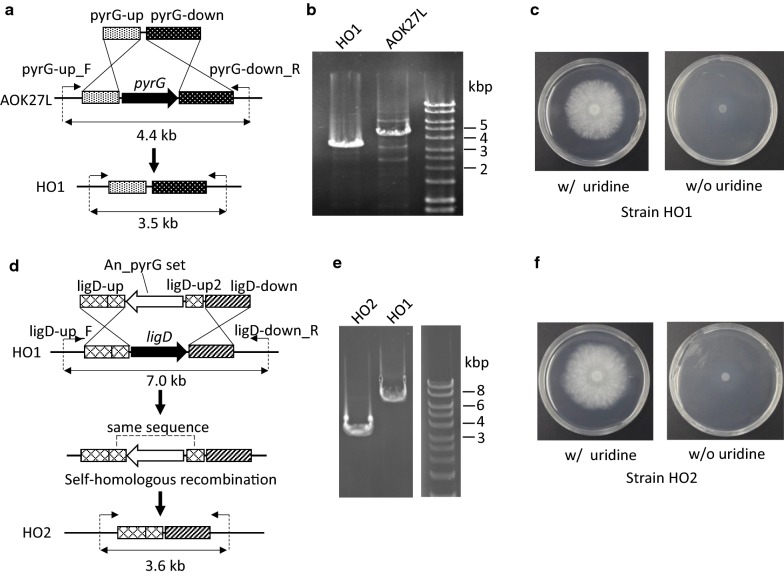
Construction of a platform strain for recombinant enzyme production by deleting *pyrG* and *ligD* of *Aspergillus oryzae* strain AOK27L. **a** Schematic of *pyrG* deletion. The resulting strain was named *A. oryzae* strain HO1. **b** PCR assay confirming *pyrG* deletion. The primer set used and the target sizes of amplicons with and without *pyrG* are shown in **a**. **c***A. oryzae* strain HO1 on a CD agar plate with and without uridine, showing uridine auxotrophy. **d** Schematic of *ligD* deletion. The resulting strain was named *A. oryzae* strain HO2. **e** PCR assay confirming *ligD* gene deletion. The primer set used and the target sizes of amplicons with and without *ligD* are shown in **d**. **f***A. oryzae* strain HO2 on a CD agar plate with and without uridine, showing uridine auxotrophy. *PCR* polymerase chain reaction

First, we introduced a DNA insert into the strain AOK27L to delete *pyrG* by homologous recombination of the flanking regions (Fig. 4a). Several colonies grew on a selection plate containing 5-fluoro-orotic acid (5-FOA) and uridine, where it was expected that the WT strain would be unable to grow, whereas *pyrG*-deleted mutants would grow normally [47]. We selected one transformant in which the deletion of the *pyrG* gene was confirmed by PCR (Fig. 4b). We named it *A. oryzae* strain HO1. It was demonstrated that the strain had uridine auxotrophy by comparative culture analysis of its growth on a CD agar plate with and without uridine (Fig. 4c). Establishment of the uridine auxotrophic strain enabled positive screening of transformants using 5-FOA in subsequent experiments.

Next, we performed *ligD* deletion in the strain HO1 by introducing a DNA fragment that contained the *pyrG* gene from *A. nidulans* flanked by *ligD*-adjacent regions designed to promote homologous recombination (Fig. 4d). We obtained 36 colonies by plate selection using CD agar without uridine. Of these, four strains contained the intended DNA fragment, which was determined by PCR. We then prepared a spore solution of one strain and inoculated it on a CD agar plate containing 5-FOA and uridine to remove the foreign *pyrG* gene from the locus by homologous recombination between the identical regions located on both ends of the *ligD* gene. We successfully obtained a colony whose PCR amplicon indicated the lack of the *pyrG* insert (Fig. 4e). Moreover, we confirmed that the strain was auxotrophic for uridine by comparative culture analysis (Fig. 4f). We named this strain *A. oryzae* strain HO2.

It has been reported that the deletion of *ligD* gene in the *A. oryzae* strain NS4 improved gene-targeting efficiency [37]. A comparison of *A. oryzae* strains HO1 and HO2 confirmed a dramatic increase in the gene-targeting efficiency ascribed to *ligD* deletion, from 11% to 97% (Table 3).

**Table 3:** Frequency of homologous integration before and after *ligD* deletion

Strain (*ligD* gene)	Obtained transformants	Homologous integrants	Gene-targeting efficiency (%)
HO1 (+)	36	4	11
HO2 (−)	39	38	97

**Fig. 5:**
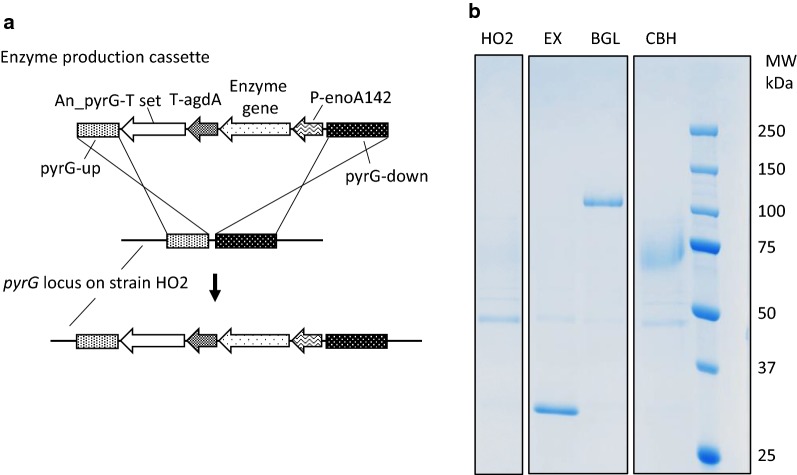
Heterologous saccharification enzyme production using *Aspergillus oryzae* strain HO2. **a** Introduction of the enzyme production cassette into the original *pyrG* locus. **b** SDS-PAGE for liquid cultures of *A. oryzae* strain HO2 and the three transformed strains possessing three respective genes: *EX* from *Talaromyces aurantiacus* (calculated molecular weight: 34 kDa), *BGL* from *T. cellulolyticus* (83 kDa), and CBH from *T. cellulolyticus* (52 kDa). SDS-PAGE, sodium dodecyl sulfate-polyacrylamide gel electrophoresis; *EX* endoxylanase, *BGL* β-glucosidase, *CBH* cellobiohydrolase

For strain HO1, data were obtained from the integration of the *ligD* deletion cassette (Fig. 4d). For strain HO2, data were obtained from the integration of the CBH production cassette (Fig. 5a)

### Heterologous saccharification enzyme production using the *A. oryzae* HO2 strain

Finally, we determined if the resulting strain HO2 was able to produce and secrete exogenous saccharifying enzymes. We tested the integration of three genes, endoxylanase (EX) from *Thermoascus aurantiacus* and β-glucosidase (BGL), and cellobiohydrolase (CBH), from *T. cellulolyticus* (Fig. 5a). The expression cassettes containing the *enoA142* promotor derived from the *A. oryzae* strain OZ (Ozeki Corporation, Hyogo, Japan; [25]), the terminator region of α-glucosidase (*agdA*) from the *A. oryzae* strain HO2, and the respective enzyme genes were introduced into the original *pyrG* locus of the strain HO2. SDS-PAGE analyses of the liquid cultures of the resulting transformants successfully demonstrated that the strain HO2 was capable of producing all the three enzymes (Fig. 5b). Their apparent (from SDS-PAGE) to calculated (from amino acid sequences excluding estimated signal peptides) molecular weights (kDa) were 31 to 34 for EX, 105 to 83 for BGL, and 70 to 52 for CBH. For recombinant CBH, the specific activity per weight for reagent cellulose (Avicel) was confirmed to be comparable with that of the native enzyme: 0.46 U/mg (standard deviation of triplicate: 0.06) of recombinant to 0.51 U/mg (0.05) of native. The results indicated that *A. oryzae* strain HO2 can serve as a platform strain to produce exogenous saccharifying enzymes and to be used in on-site SSF recombinant enzyme production.

## Discussion

One major technological challenge in the proposed on-site SSF recombinant enzyme production system is fermentation inhibition due to chemical components ascribed to biomass pretreatment [48]. The negative effects of chemicals (e.g., furfural, HMF, and acetic acid), originating from processed, especially dilute sulfuric acid-treated, biomass on ethanol fermentation by yeast have been extensively studied [49–51]. It would be reasonable to anticipate a similar phenomenon in the case of protein production by fungi. In addition, because of a higher aqueous concentration of inhibitors, SSF potentially imposes severe conditions compared with the conventional LSF. The amount of fermentation inhibitors in ammonia-treated biomass were significantly lower than that in dilute sulfuric acid–treated biomass (Table 1). This trend has also been confirmed in comparison with dilute sulfuric acid-treated rice straw. This finding supports the advantage of ammonia-treated biomass to be used as a nutrient for enzyme production under SSF conditions.

The strains industrially used *A. oryzae strains* for *shoyu* and *miso* fermentation tended to grow better under SSF conditions compared with strains used for *sake* brewing except *A. oryzae* strain AOK2P (Table 2, Fig. 2a, and Additional file 1: Fig. S1). In SDS-PAGE analysis (Fig. 2b), the distinct band around 50 kDa, which was pervasive for all the tested strains in this study, can be α-amylase [52, 53]. The strains industrially used for *shoyu* and *miso* fermentation secrete various proteins, in addition to α-amylase, compared with strains used for *sake* brewing. Since *shoyu* and *miso* are made from mixed grains (e.g., soybean and wheat), unlike *sake*, which is made from polished rice grains, the employed strains likely secrete cellulases and hemicellulases to saccharify the raw ingredients. Several studies suggest that cellulases and hemicellulases, including xylanases, are produced by *A. oryzae* strains grown on soybeans [54, 55]. The identification of these secreted proteins and the investigation of their enzymatic activities may provide useful information that is applicable to biomass saccharification in the biorefinery field. At the same time, there is a possibility that proteases are also secreted by the strains industrially used in *shoyu* and *miso* fermentation [56]. These enzymes could hydrolyze and deactivate necessary saccharifying enzymes, and, therefore, hinder the SSF. In these cases, the deletion of the identified protease genes would be a direct and effective approach to counteract this issue.

In this study, we selected the strain AOK27L as a wild-type strain from which we developed a platform for targeted recombinant enzyme production. In this study, the strain selection was performed based on the following two criteria: i) the visibility of mycelium and ii) the concentration of total secreted proteins. Of note, there is another criterion that can be used for strain selection, which is the amount of secreted proteins per weight of the cells growing on the biomass. Our results showed that the strains without visible mycelium, i.e., the strains RIB40, AOK20, AOK65, and AOK241, actually grew on the pretreated biomass, which was confirmed by the coagulation of biomass particles and the noticeable amount of secreted protein (Fig. 2b, c). In the total secreted protein assay, the strains AOK27L, RIB40, and AOK65 were the first, second, and third most active producers, respectively. If we measure the amount of secreted proteins per weight of the cells growing on the biomass, the strains RIB40 and AOK65 likely show higher values than the strain AOK27L. To use this index, it is necessary to measure the weight of vegetative cells in SSF. However, as the mycelium tightly binds onto or penetrates biomass particles, and it is virtually impossible to separate them from each other, the weight of vegetative cells cannot be measured directly [57]. The quantification of glucosamine derived from chitin, a component of the cell wall, by acid hydrolysis could serve as a surrogate [58].

Zhang et al. [46] studied the effects of acetic acid, formic acid, vanillin, HMF, and furfural on the yield of gluconic acid fermentation by *A. niger*. Despite several different experimental setups (i.e., product of fermentation, solid-/aqueous-state fermentation, and the *Aspergillus* strain), their results provided us with good guidance to assess the effects of the five chemicals as fermentation inhibitors. Referring to their data, of the five fermentation inhibitors, acetic acid could have a slight inhibitory effect, but the others are within the safe concentration range where no fermentation inhibition is anticipated.

In contrast to Zhang et al.’s [46] and Casey et al.’s [59] studies on ethanol-fermenting yeasts, up to 7.9 g/L (132 mM) of acetic acid exerted a salient enhancing effect on the protein production by both *A. oryzae* strains AOK27L and RIB40 (Fig. 3). With regard to the effects of acetic acid, one parameter which we should consider is the pH of the medium. Acetic acid in an undissociated state under acidic conditions that is liposoluble can diffuse into microbial cells across the plasma membrane and inhibit growth by decreasing the cytosolic pH [60]. The undissociated acid concentration is a function of p*K*a (negative log of the acid dissociation constant) and pH of the medium, and increases with decreasing pH according to the Henderson–Hasselbalch equation [61]. The optimum pH values for *Aspergillus* species growth are ~ 6.0, which was the value adopted by us and Zhang et al. [46]. In contrast, ethanol-fermenting yeasts (e.g., *Saccharomyces cerevisiae*) grow optimally in the pH range of 5.0–5.5 [60]. Therefore, when comparing the effects of acetic acid on two different groups of fermenting microorganisms at their respective optimum pH, *Aspergillus* species benefit from their higher optimum pH and the consequent lower undissociated acid concentration. Taking 4.75 as the p*K*a of acetic acid (at 0 ionic strength and 25 °C [62]), the ratio of undissociated acid to the total acid (undissociated + dissociated) is calculated to be 0.053 at pH 6.0 and 0.36 at pH 5.0.

The mechanism by which acetic acid increased protein production in our LSF experiment remains unclear. The enhancement of up to 4.7-fold for the strain AOK27L on the addition of 7.9 g/L acetic acid could not be accounted for just by the additional amount of carbon nutrient as PD medium was already carbon-rich, containing 2% dextrin hydrate, 1% polypeptone peptone, and 0.1% casamino acid. Klein et al. [63] recently reported that acetate-containing substrate mixtures improve recombinant protein secretion in *Schizosaccharomyces pombe* by 1.8- and 3.8-fold for green fluorescent protein (GFP) and a GFP-coupled antibody fragment, respectively. They proposed that the addition of acetate and the consequent increase in cellular lipid content allows for increased membrane and vesicle formation, which can improve the transport and secretion of otherwise intracellularly retained recombinant proteins. Our finding that acetic acid, which is considered a potential fermentation inhibitor, can actually act as a strong enhancer of protein production by *A. oryzae* has far-reaching implications for the entire biorefinery field, since acetic acid can inevitably be derived from thermochemically processed biomass [64]. Understanding the underlying mechanisms would lead to the development of a new cellular engineering technology for enhanced protein production and other biomass-based fermentation processes [65]. One promising future direction would be to combine genome, transcriptome, and metabolome analyses [66, 67]. Since the genomic sequences of both the *A. oryzae* strains used in this study are known, we would be able to identify candidate genes related to the transport and secretion of proteins. Furthermore, the comparison of transcriptome and/or metabolome data obtained in the culture conditions with and without acetic acid would facilitate the elucidation of some potentially key genes that behave dependently on the culture condition and of their functions.

Uridine auxotrophic strains have been isolated by ultraviolet radiation or mutagenic chemicals [68, 69], but these methods might introduce undesirable mutations into genomic DNA. Comparatively, since homologous recombination can excise only *pyrG*, the effect on genomic DNA can be suppressed. The improvement of gene-targeting efficiency by *ligD* deletion used the marker-recycling method in which self-homologous recombination occurs, and we could confirm the applicability of the previously reported method [70] (Fig. 4d–f) and also the drastic effect of *ligD* deletion on the gene-targeting efficiency (Table 3).

In the SDS-PAGE analysis (Fig. 5b), the apparent molecular weights of recombinant BGL and CBH were larger compared with the calculated values. In addition, the band of recombinant CBH was rather broad. These results can be attributed to N-type glycosylation [17, 71]. N-type glycosylation has been observed with the other recombinant proteins produced by *A. oryzae* [72]. It reportedly contributes to the heat resistance of enzymes [73]. Our finding that strains producing recombinant proteins reduce α-amylase secretion (the band ~ 50 kDa) compared with untransformed *A. oryzae* strain HO2 should be titration effect of the *cis*-regulatory element possessed in the *enoA142* promoter used for enzyme expression [74].

In the future, we will use the strains developed in this study to explore the possibility of recombinant enzyme production under SSF conditions. The key milestones from a practical point of view will be how to produce multiple enzymes in a single batch, control their production ratio, scale up the fermentation system, control the production ratio of saccharifying enzymes, and demonstrate biomass saccharification. Large-scale SSF apparatus for growing *A. oryzae* on solid grains during *shoyu* and *miso* production is already well established in the food industry [75]. An analogy would provide valuable information and expedite the development of biomass SSF system.

## Conclusions

It is feasible to use *A. oryzae* as a host microorganism for on-site SSF recombinant enzyme production using ammonia-treated rice straw as model biomass. The *A. oryzae* strains industrially used to produce *shoyu* and *miso* grow better under test conditions. Acetic acid, which is concomitant with ammonia-treated rice straw, improves protein production by *A. oryzae* under LSF conditions. We also constructed heterogeneous enzyme-producing strains on the basis of the selected strain and achieved secretory production of three saccharifying enzymes. *A. oryzae* is a promising candidate as a host microorganism for on-site SSF recombinant enzyme production, and the *A. oryzae* strain developed in this study will be a powerful platform strain for secretory production of heterologous saccharifying enzymes using the cost-effective on-site SSF recombinant enzyme production system.

## Methods

### *A. oryzae* strains and culture medium

*Aspergillus oryzae* strain RIB40 was obtained from the National Research Institute of Brewing (Hiroshima, Japan), and seven other strains were purchased from Akita Konno Co., Ltd. (Daisen, Japan). *A. oryzae* cultures were maintained using PD medium: 2% (w/v) dextrin hydrate, 1% (w/v) polypeptone peptone, 0.1% (w/v) casamino acid, 0.5% (w/v) KH_2_PO_4_, 0.1% (w/v) NaNO_3_, and 0.05% (w/v) MnSO_4_·4H_2_O at pH 6.0. For sporulation and screening of gene transformants, plate culture was conducted on CD agar medium, containing 3% (w/v) dextrin hydrate, 0.2% (w/v) KCl, 0.1% (w/v) KH_2_PO_4_, 0.3% (w/v) NaNO_3_, 0.05% (w/v) MnSO_4_·4H_2_O, and 0.001% (w/v) FeSO_4_·7H_2_O at pH 6.0. Spores resulting from 1-week cultivation were collected with 0.01% (v/v) Tween 20.

### Biomass pretreatment

Dry rice straw was ground, passed through a 3 mm mesh, and mixed with four times its weight of aqueous ammonia (25%, w/w). The mixture was kept for 8 h at 80 °C and then dried for 1 day at 50 °C to evaporate water and ammonia [17]. The resulting substrate was kept dry in a desiccator until use in downstream experiments.

The glucan and xylan contents of pretreated biomass were determined according to the standard methods given by the NREL (CO, USA) [76]. To assess the fermentation inhibitor content, 20% (w/v) of the pretreated biomass was hydrolyzed using commercially available Cellic Ctec2 cellulase (Novozymes, Denmark) at a dosage of 1.3 mL/g of dry biomass at 50 °C and pH 5.0 for 72 h. After centrifugation at 5000×*g* for 10 min, the supernatant of the resulting slurry was subjected to high-performance liquid chromatography (HPLC) assays using an Aminex HPX-87H column (Bio-Rad Laboratories, Hercules, CA, USA) for organic acids and a Shim-pack VP-ODS column (Shimadzu Corporation, Kyoto, Japan) for aromatic compounds. For each fermentation inhibitor, the measured concentration was divided by the pretreated biomass concentration at hydrolysis to obtain the composition (mg/g of pretreated biomass), as described previously [46].

### SSF validation test

A fraction of the pretreated biomass retained between sieves of 0.5 and 1 mm mesh was mixed with the same weight of purified water (Milli-Q: Merck Millipore, Burlington, Massachusetts, United States). The pH was adjusted to 6 using 2 M HCl. For each *A. oryzae* strain, 5 g of the wet substrate was autoclaved for 15 min at 121 °C and, after cooling, it was mixed with the spore suspension (1 × 10^6^ spores). The water content was measured using a Shimadzu moisture MOC-12H analyzer. Then, the inoculated substrate was transferred to a Petri dish and incubated for 40 h at 95% relative humidity and 30 °C in an environmental chamber (EYELA, Japan), and then, the substrate was washed with 15 mL of purified water and centrifuged for 10 min at 10,000×*g*. The supernatant was filtered through a 0.22 μm Durapore membrane (Millipore, Burlington, MA, USA). Quantification of the protein amount was performed using Protein Assay Coomassie Brilliant Blue (CBB) Solution (Nacalai Tesque, Kyoto, Japan), an absorbance microplate reader (Hitachi, Tokyo, Japan), and bovine serum albumin (Nacalai Tesque) as the calibration standard.

For SDS-PAGE, precast gels and preformed, running CBB staining buffer solutions were obtained from Atto Corporation (Japan). A prestained protein standard was purchased from Bio-Rad. Quantification of corresponding bands on the SDS-PAGE gel image to estimate the target enzyme concentrations was performed using a Bio-Rad imager [77]. Calibration for the quantification of each target protein was conducted using the same software.

### Liquid fermentation with and without acetic acid

For each *A. oryzae* strain, 50 mL of PD medium was prepared with and without acetic acid (final concentration 5.3 or 7.9 g/L). The pH was adjusted to 6 using 2 M HCl (without acetic acid) or NaOH (with acetic acid). Spore suspensions of *A. oryzae* strains RIB40 and AOK27L were inoculated in the medium (final concentration 1 × 10^4^ spores/mL) and incubated for 72 h at 30 °C. The supernatants were filtered through a 0.22 μm Durapore membrane (Millipore). Quantification of the protein amount was performed using Protein Assay CBB Solution (Nacalai Tesque), as described before.

### Genome sequencing

Paired-end reads of *A. oryzae* strain AOK27L were generated using HiSeq 2500 (Illumina, San Diego, CA, USA) and mapped to *A. oryzae* strain RIB40 sequences using Burrows–Wheeler Alignment Tool ver. 0.6.2. The operations were performed by Genaris, Inc. (Yokohama, Japan). Using the resulting sequence data, we designed the primers for this study.

### Construction of plasmids and *A. oryzae* transformation

**Fig. 6:**
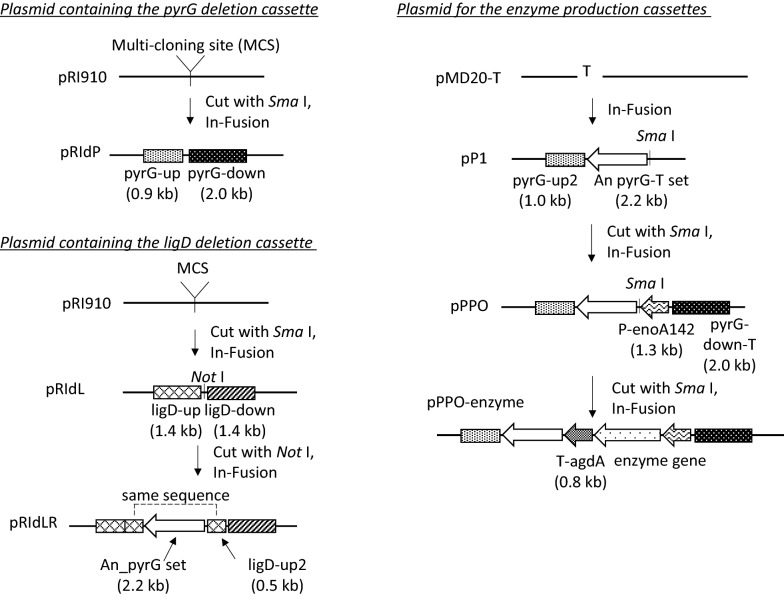
Procedures for the preparation of plasmid constructs used in this study

The procedures for preparing the plasmid constructs used in this study are summarized in Fig. 6. The genes amplified by PCR were integrated on plasmids using In-Fusion technology [78]. An Applied Biosystems thermal cycler and KOD Plus polymerase (Toyobo Co., Osaka, Japan) were used for all PCR experiments. The PCR reaction mixture and polymerase conditions were as described in the user manual. Genomic DNAs were purified as templates using a MasterPure Yeast DNA Purification kit (Lucigen Corporation, Middleton, WI, USA) from the following fungal strains: *A. oryzae* strain AOK27L, *A. oryzae* strain OZ, *A. nidulans* strain FGSC-A4 [79], *T. cellulolyticus* strain H1 [15], and *T. aurantiacus* strain SG [80]. Table 4 shows the list of primer pairs and templates used, which were purchased from Eurofins Genomics K.K. (Tokyo, Japan). Plasmids, restriction enzymes, and an In-Fusion HD Cloning Kit were obtained from Takara Bio Inc. (Kusatsu, Japan).

**Table 4:** PCR primers and templates used to prepare plasmid constructs

Primer pair	Sequence (5′ to 3′)	Template
*PyrG disruption cassette*		
pyrG-up	tcgagctcggtacccccagaggtgactttatccaagattcc	AOK27L
	cccgggcaattgccgcgaaaaattaaattgaatc	
pyrG-down	cggcaattgcccggggtagtggtggatacgtactccttttatg	AOK27L
	ctctagaggatccccttcaggtcacgttctaagcttatcagc	
*LigD deletion cassette*		
ligD-up	tcgagctcggtacccggttactgctctcccttgatgatg	AOK27L
	taggtagtgaacctatttcgagagcag	
ligD-down	taggttcactacctagcggccgcacaggcaccttgcatcatcatc	AOK27L
	ctctagaggatccccggaccgacgattcgttgaagag	
ligD-up2	acaggtatcgaattcgtccttgtgacgacgagctcg	AOK27L
	ggtgcctgtgcggccgctaggtagtgaacctatttcgagagcag	
An_pyrG set	gaattcgatacctgtcgaaagaaatggaag	FGSC-A4
	cactacctagcggcctcagtgcttgtctaccagattagggag	
*Enzyme production cassettes*		
pyrG-up2	actagtcatatggatccagaggtgactttatccaagattcc	AOK27L
	gtagacaagcactgacaattgccgcgaaaaattaaattgaatctatgg	
An_pyrG-T set	acccggggatccgatgaattcgatacctgtcgaaagaaatggaag	FGSC-A4
	Tcagtgcttgtctaccagattaggg	
pyrG-down-T	gacagtcgtcgttgggtagtggtggatacgtactccttttatgg	AOK27L
	tcgagctcggtacccttcaggtcacgttctaagcttatcagc	
P-enoA142	ccaacgacgactgtctcattactagtc	OZ
	attcatcggatcccgggtttgcgagtggtttggtaaaaggtag	
T-agdA	gaagcgtaacaggatagcctagacc	AOK27L
	aattcatcggatcccagtaacccattcccggttctctag	
cbh	accactcgcaaacccatgtctgccttgaactctttcaatatgtacaag	H1
	atcctgttacgcttcctacaaacattgagagtagtaagggttcacg	
bgl	accactcgcaaacccatgtactccgcctttctgctc	H1
	atcctgttacgcttctcactggaggcactgggag	
ex	accactcgcaaacccatggtccgccctaccatcc	SG
	atcctgttacgcttctcactgctggagatcctggac	

Templates are genomic DNAs purified from the indicated strains. AOK27L: *A. oryzae* strain AOK27L, FGSC-A4: *Aspergillus nidulans* strain FGSC-A4, OZ: *A. oryzae* strain OZ, H1: *T. cellulolyticus* strain H1, SG: *T. aurantiacus* strain SG

To prepare transformation inserts, DNA fragments were amplified by PCR using template plasmids and primer sets (Table 5) and purified using the QIAquick PCR Purification Kit (QIAGEN, Hilden, Germany). *A. oryzae* strain AOK27L transformation was performed, as described previously [81], and colonies of transformants were selected on CD agar medium. For screening of *pyrG*-deleted mutants, CD agar was supplemented with 0.1% (w/v) 5-FOA and 0.5% (w/v) uridine.

**Table 5:** PCR templates and primers used to prepare transformation inserts

Primer pair	Sequence (5′ to 3′)	Template
*PyrG or ligD deletion cassettes*		
pRI	gcaattaatgtgagttagctcactc	pRIdP or pRIdLR
	ggatatcggggaagaacagtatgtc	
*Enzyme production cassettes*		
pMD20c	cagtgagcgcaacgcaattaatgtgagttag	pPPO enzyme
	gggatgtgctgcaaggcgattaagttg	

Templates are the plasmid constructs prepared in this study (Fig. 6)

### Production and activity assay of recombinant enzymes

The three enzymes tested were CBH (GenBank accession no. GAM33347) and BGL (GenBank accession no. GAM40530) from *T. cellulolyticus* and EX (GenBank accession no. AAF24127) from *T. aurantiacus*. Whereas *CBH* was cloned from genomic DNA, *BGL* and *EX*, were prepared as codon-adjusted synthetic DNAs by Takara Bio Inc. (sequence data in Additional file 2: Fig. S2). Three transformed *A. oryzae* HO2 strains, each of which possessed the respective gene, were grown in PD medium for 7 days at 30 °C. The culture broth was filtered through a 0.22 μm Durapore membrane (Millipore), and target enzyme production in the filtrates was assayed by SDS-PAGE, as described before. The molecular weights of the three recombinant proteins were calculated using GENETYX version 12.0 (Genetyx Co., Tokyo, Japan) based on the amino acid sequences excluding the signal sequences that were predicted using SignalP 5.0 server [82].

Native CBH was obtained by liquid culture of *T. cellulolyticus* strain H1 and serial column chromatography, as described previously [16]. Activity assay and deglycosylation for CBH were performed, as described previously [17].

## Supplementary information

**Additional file 1: Fig. S1.** Images of biomass inoculated with the strains tested in the validation test.

**Additional file 2: Fig. S2.** Sequences of *BGL* from *Talaromyces cellulolyticus* and *EX* used in this study. *BGL*, β-glucosidase; *EX*, endoxylanase.

## Data Availability

The genomic sequence data of *A. oryzae* strain AOK27L are available from the corresponding author on reasonable request. Other data generated or analyzed during this study are included in this published article and its additional files.

## Abbreviations

5-FOA: 5-Fluoro-orotic acid
BGL: β-Glucosidase
CBB: Coomassie Brilliant Blue
CBH: Cellobiohydrolase
EX: Endoxylanase
FDA: Food and Drug Administration
GFP: Green fluorescent protein
GRAS: Generally Recognized as Safe
HMF: 5-Hydroxymethylfurfural
HPLC: High-performance liquid chromatography
LSF: Liquid-state fermentation
NREL: National Renewable Energy Laboratory
PCR: Polymerase chain reaction
SDS-PAGE: Sodium dodecyl sulfate-polyacrylamide gel electrophoresis
SSF: Solid-state fermentation
WT: Wild type
