# Supplementary material for: Feasibility study of on-site solid-state enzyme production by Aspergillus oryzae
Source: Biotechnol Biofuels. 2020 Feb 26;13:31. doi: 10.1186/s13068-020-1669-3 (PMC7045521; doi:10.1186/s13068-020-1669-3)
Supplement: Supplementary file 1 — Additional file 1: Fig. S1. Images of biomass inoculated with the strains tested in the validation test. [file 13068_2020_1669_MOESM1_ESM.pptx]

## Slide 1
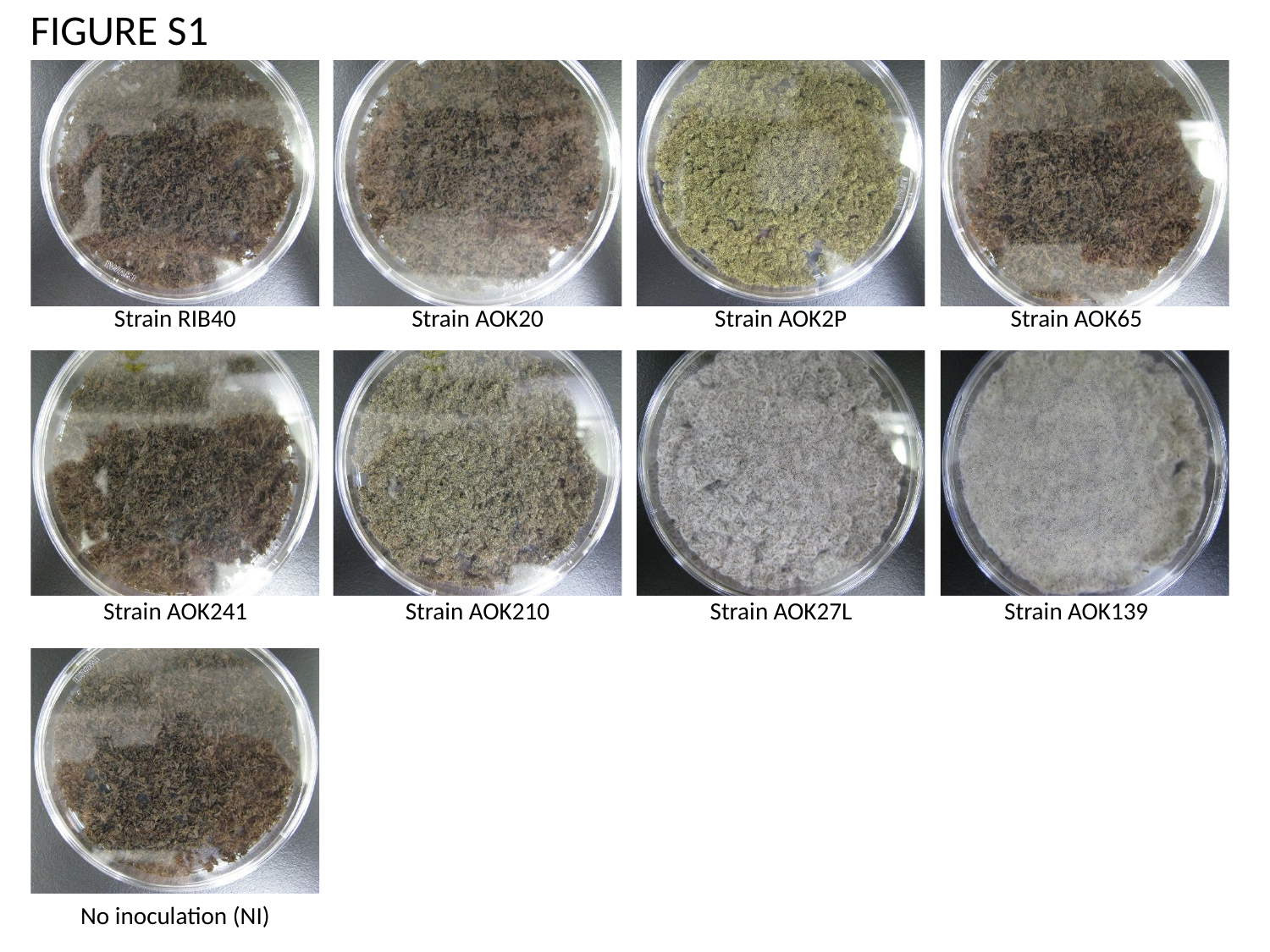

FIGURE S1
Strain RIB40
Strain AOK20
Strain AOK2P
Strain AOK65
Strain AOK241
Strain AOK210
Strain AOK27L
Strain AOK139
No inoculation (NI)
