# Supplementary material for: Feasibility study of on-site solid-state enzyme production by Aspergillus oryzae
Source: Biotechnol Biofuels. 2020 Feb 26;13:31. doi: 10.1186/s13068-020-1669-3 (PMC7045521; doi:10.1186/s13068-020-1669-3)
Supplement: Supplementary file 2 — Additional file 2: Fig. S2. Sequences of BGL from Talaromyces cellulolyticus and EX used in this study. BGL, β-glucosidase; EX, endoxylanase. [file 13068_2020_1669_MOESM2_ESM.pptx]

## Slide 1
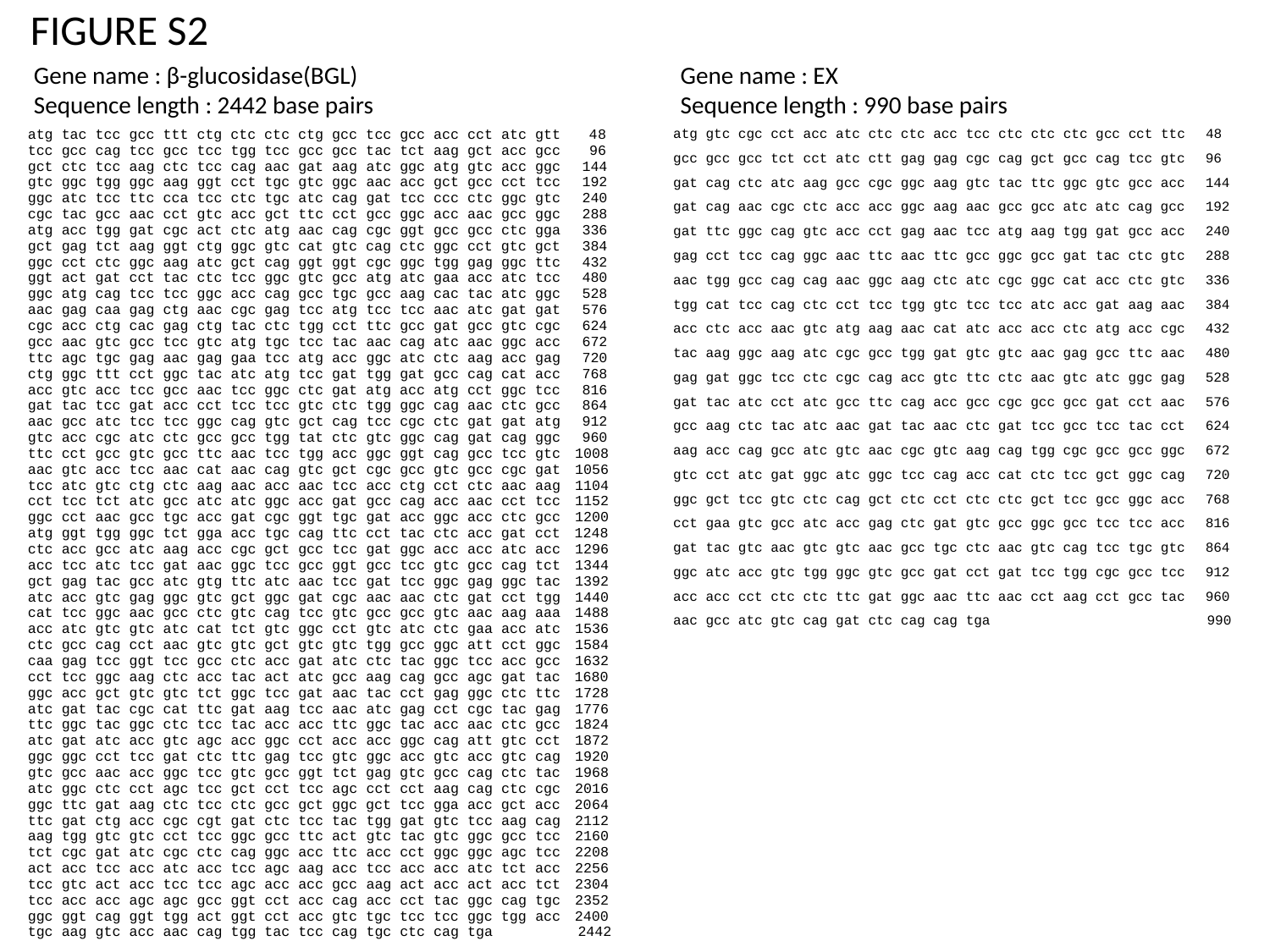

FIGURE S2
Gene name : β-glucosidase(BGL)
Sequence length : 2442 base pairs
Gene name : EX
Sequence length : 990 base pairs
